# Supplementary material for: Impact of Modifiable Factors Associated with Physical Frailty and Cognitive Impairment Trajectory of Older Adults: Using the Korean Longitudinal Study of Aging 2006–2018
Source: Healthcare (Basel). 2025 Feb 4;13(3):315. doi: 10.3390/healthcare13030315 (PMC11817315; doi:10.3390/healthcare13030315)
Supplement: Supplementary file 1 [file healthcare-13-00315-s001.zip › healthcare-3404634-supplementary.pdf]

**Supplementary**

**Table S1.** The GRoLTS checklist in the study

| No. | Checklist item | Reported? |
|---|---|---|
| 1. | Is the metric of time used in the statistical model reported? | Yes |
| 2. | Is information presented about the mean and variance of time within a wave? | Yes |
| 3a. | Is the missing data mechanism reported? | No |
| 3b. | Is a description provided of what variables are related to attrition/missing data? | No |
| 3c. | Is a description provided of how missing data in the analyses were dealt with? | Yes |
| 4. | Is information about the distribution of the observed variables included? | Yes |
| 5. | Is the software mentioned? | Yes |
| 6a. | Are alternative specifications of within-class heterogeneity considered (e.g., LGCA vs. LGMM) and clearly documented? If not, was sufficient justification provided as to eliminate certain specifications from consideration? | No |
| 6b. | Are alternative specifications of the between-class differences in variance–covariance matrix structure considered and clearly documented? If not, was sufficient justification provided as to eliminate certain specifications from consideration? | N/A |
| 7. | Are alternative shape/functional forms of the trajectories described? | Yes |
| 8. | If covariates have been used, can analyses still be replicated? | N/A |
| 9. | Is information reported about the number of random start values and final iterations included? | Yes |
| 10. | Are the model comparison (and selection) tools described from a statistical perspective? | Yes |
| 11. | Are the total number of fitted models reported, including a one-class solution? | Yes |
| 12. | Are the number of cases per class reported for each model (absolute sample size, or proportion)? | Yes |
| 13. | If classification of cases in a trajectory is the goal, is entropy reported? | Yes |
| 14a. | Is a plot included with the estimated mean trajectories of the final solution? | Yes |
| 14b. | Are plots included with the estimated mean trajectories for each model? | N/A |
| 14c. | Is a plot included of the combination of estimated means of the final model and the observed individual trajectories split out for each latent class? | N/A |
| 15. | Are characteristics of the final class solution numerically described (i.e., means, SD/SE, *n*, CI, etc.)? | Yes |
| 16. | Are the syntax files available (either in the appendix, supplementary materials, or from the authors)? | Yes |

*Note*: Adopted from the article of van De Schoot et al. (2017).
Abbreviations: CI, confidence interval; GRoLTS, Guidelines for Reporting on Latent Trajectory Studies; LGCA, latent class growth analysis; LGMM, latent growth mixture modeling; N/A, not applicable; SD, standard deviation; SE, standard error.
